# Impact of care coordination on oral anticoagulant therapy among patients with atrial fibrillation in routine clinical practice in Japan: a prospective, observational study

**DOI:** 10.1186/s12872-019-1216-y

**Published:** 2019-10-24

**Authors:** Fumiko Ono, Sayako Akiyama, Akifumi Suzuki, Yoshinobu Ikeda, Akira Takahashi, Hitoshi Matsuoka, Masahiro Sasaki, Tomonori Okamura, Nariaki Yoshihara, The Akita study group, Akira Takahashi

**Affiliations:** 1 Health Economics & Outcomes Research, Bayer Yakuhin, Ltd., 6-5 Marunouchi 1-chome, Chiyoda-ku, Tokyo, 100-8265 Japan; 2 Local Independent Administrative Institution, Akita Prefectural Hospital Organization, Akita, Japan; 3 The Japan Stroke Association, Osaka, Japan; 4 Ikeda Clinic, Akita, Japan; 5 Takahashi Internal Medicine Clinic, Akita, Japan; 6 Matsuoka Internal Medicine Clinic, Akita, Japan; 7 Research Institute for Brain and Blood Vessels-Akita, Akita, Japan; 8 Department of Preventive Medicine and Public Health, Keio University School of Medicine, Tokyo, Japan

**Keywords:** Atrial fibrillation, Care coordination, Anticoagulants, Aging society, Regional medical care, Opportunistic screening in primary care

## Abstract

**Background:**

Care coordination between general practitioners (GPs) and cardiovascular specialists is expected to play a key role in establishing appropriate oral anticoagulant (OAC) treatment in atrial fibrillation (AF) patients. The aim of this study was to assess the impact of care coordination on oral anticoagulant therapy in the management of AF in Japan.

**Methods:**

This study was a multi-center, single-arm, prospective cohort study with retrospective chart and claims data review for historical controls. The study included three study periods: a 12-month pre-campaign period; a 12-month campaign period for AF screening and care coordination; and a 3-month post-campaign period for follow-up of care coordination. During the campaign period, patients aged ≥65 years who attended participating GP clinics underwent opportunistic AF screening by GPs under the campaign. At the discretion of the GP, newly diagnosed AF patients after the screening were referred to a cardiovascular specialist for care coordination. To assess the impact of care coordination and evaluate the effects of the campaign, implementation of care coordination, antithrombotic therapies, and patient-reported outcomes were compared between patients with and without care coordination, and between patients during the pre-campaign and campaign periods.

**Results:**

There were 86 newly diagnosed AF patients during the pre-campaign period and 90 during the campaign period. The percentage of patients with care coordination increased from 3.5% (3/86) in the pre-campaign period to 14.4% (*n* = 13/90) during the campaign period. The percentage of patients who received OAC therapies, according to the definition from the Japanese AF medication guideline, increased from 55.8% (48/86) to 71.1% (64/90) during the campaign period regardless of care coordination. Younger patients were referred to cardiovascular specialists for care coordination. Implementation of OAC therapy did not differ between patients with and without care coordination. Adherence to OAC therapy was low regardless of care coordination.

**Conclusions:**

This GP-targeted campaign was effective at raising awareness regarding the implementation of care coordination and appropriate OAC therapy at local clinical practices in Japan. Improvement of adherence to OAC therapy in elderly patients is a critical issue, and measures such as education programs targeted to patients and healthcare professionals should be undertaken.

## Background

Atrial fibrillation (AF) is a common arrhythmia with a poor prognosis in elderly populations [1, 2], and its prevalence increases with age [3, 4]. Given the continuing growth of the aging population in Japan, an estimated 1 million patients are expected to have AF by 2030 [5]. Patients with AF have a high risk of cardioembolic stroke and thromboembolic complications, up to five times that of a person in sinus rhythm [6–8], and they have a 1.5- to 1.9-fold higher risk of mortality [9]. Moreover, AF is now considered the second most important risk factor for stroke, causing one in five strokes [10, 11], and cardioembolic strokes in patients with AF are more severe than those in patients without AF [7].

In Japanese populations, previous studies have identified AF as an independent and major risk factor for stroke and have found an association between AF and early death in this population [6, 12]. Although appropriate anticoagulant treatment for the prevention of cardioembolic stoke is needed for patients with AF, in particular elderly patients [13, 14], studies of oral anticoagulant (OAC) use in Japan have reported that around half of patients either do not receive OAC treatment or they receive inappropriate OAC treatment [15, 16]. An international systematic review from 2010 also reported that about half of patients requiring OACs were not treated [17]. On the other hand, appropriate OAC treatment in patients with AF has been demonstrated to prevent the risk of stroke and subsequent morbidity and mortality [15, 18, 19].

Care coordination between general practitioners (GPs) and cardiovascular specialists is expected to play a key role in establishing appropriate OAC treatment in patients with AF. The European Society of Cardiology (ESC) guidelines recommend the implementation of integrated AF management, including care coordination, between GPs and cardiovascular specialists for AF patient management [18]. Nonetheless, the effectiveness of care coordination in real-life settings in AF management has not been established; however, it is expected that care coordination will improve outcomes and overcome issues such as underuse of anticoagulants and inconsistent patient management [18, 20, 21].

Therefore, this study aimed to investigate the effectiveness of opportunistic AF screening in patients aged 65 years and older in the primary care setting in Japan. The study also aimed to assess the impact and feasibility of care coordination between GPs and cardiovascular specialists in OAC treatment for AF patients, as well as the adherence to, benefit from, and burden of OAC treatment, and satisfaction with care coordination. This report focuses on the results of implementation of care coordination and its effect on antithrombotic therapy.

## Methods

### Study design and setting

This study was a multi-center, single-arm, prospective cohort study with retrospective chart and claims data review for historical controls. The campaign in this study was performed at 12 primary care clinics in Daisen and Yokote cities, Akita Prefecture, Japan (Additional file 1).

This study was designed as an awareness campaign targeted at GPs for screening of AF and subsequent implementation of care coordination and optimal anticoagulant therapy. In this study, a patient who received care coordination was defined as one who was referred from a GP to a cardiovascular specialist (that is, a board-certified member of The Japanese Circulation Society) and was then referred back to the GP after consultation by the specialist. In care coordination, cardiovascular specialists performed evaluation of the underlying disease, assessed the risk of stroke (CHADS2 score), and made treatment decisions if necessary. This process is within the routine medical care for AF, and primary care-centric care coordination regarding the management of AF has been introduced in some regions in Japan, in particular for further tests or procedures by cardiovascular specialists [22].

This study included three study periods: a 12-month pre-campaign period (October 19, 2014 to October 18, 2015), a 12-month campaign period (October 19, 2015 to October 18, 2016), and a 3-month post-campaign period for follow-up of referrals from a cardiovascular specialist back to the GP (October 19, 2016 to January 18, 2017). The pre-campaign period was defined as the control period without implementation of the campaign. The 12-month campaign was conducted during the campaign period to renew awareness of care coordination between GPs and cardiovascular specialists for AF management concerning stroke risk assessment and OAC treatment based on the Japanese AF medication guideline [4]. During this period, opportunistic AF screening was performed in patients aged ≥65 years who attended participating GP clinics; the screening performed during this period was more thorough and meticulous than the screening performed during the other periods. The awareness of GPs to implement care coordination during the campaign period was renewed, but they were free to implement it based on their clinical judgment, and they decided to which cardiovascular specialist to refer patients. A list of cardiovascular specialist candidates was prepared in advance to facilitate referrals, but GPs were not restricted to this list. After the assessment of risk for stroke, antithrombotic therapy for stroke prevention such as anticoagulant agents and antiplatelet agents was initiated at the discretion of the cardiovascular specialist, and patients were referred back to the GP for follow-up of ongoing therapy.

### Patients

The study population was patients with AF who were seen at one of the participating sites during the 12-month campaign period and the 12-month pre-campaign period, who were identified by claims data in each clinic. This population included outpatients aged ≥65 years who were newly diagnosed with AF during the pre-campaign or campaign periods, and those who had been previously diagnosed with AF at the study clinics prior to the start of the pre-campaign or campaign periods. For identification of patients with AF and to distinguish between newly and previously diagnosed AF patients, the claims data in each clinic were used.

In terms of patient-reported outcomes, AF patients on OAC treatment for at least 4 weeks were asked to fill out the Anti-Clot Treatment Scale (ACTS) [23] and Morisky 8-Item Medication Adherence Scale (MMAS-8) [24–26] questionnaires. Additionally, patients who received care coordination were asked to answer a patient survey on care coordination satisfaction.

Sample size estimation was decided based on feasibility, and no formal statistical sample size estimation was performed for this study. The population of elderly people aged ≥65 years was estimated to be 185,000 and 59,800 in Daisen and Yokote cities, respectively. Therefore, approximately 5000 patients from 12 participating clinics (400 patients per clinic) were expected to undergo screening during the screening period. Of them, newly diagnosed AF patients who were identified as having AF after the opportunistic screening were considered to be enrolled in the target population for the analysis in the current report.

### Variables and endpoints

The variables collected for this study included patient background data (demographic and clinical characteristics), AF diagnosis-related data (type of AF, stroke risk score (CHADS_2_ score)), treatment-related data including care coordination and antithrombotic therapies for stroke prevention (OACs including warfarin and direct OACs [DOACs], antiplatelet agents), and patient-reported data. The following endpoints for this study were descriptively compared between the pre-campaign and the campaign periods: stroke risk score (CHADS_2_ score), the number and proportion of patients who were referred to cardiovascular specialists and then back to GPs as care coordination, antithrombotic therapies for stroke prevention, antithrombotic therapies according to the definition from the Japanese guideline of AF medication [4], and patient-reported outcomes using ACTS, MMAS-8, and a care coordination satisfaction patient survey. In the current study, the definition from the Japanese guideline of AF medication includes any OAC therapy, including DOACs or warfarin, for patients with a CHADS_2_ score of 1 point or over or no OAC therapy for those with a CHADS_2_ score of 0 point. The ACTS burden total score ranges from 12 to 60, and the ACTS benefits total score ranges from 3 to 15 [23].

The above endpoints were also descriptively compared between patients who received and did not receive care coordination, except for the number and proportion of patients who were referred to cardiovascular specialists and then back to GPs as care coordination and the results for care coordination satisfaction on the patient survey.

Data of patients with AF were collected from routine medical practice. GPs entered patient data into an electronic data capture system managed under anonymous conditions. Historical patient data (demographic and clinical characteristics, AF diagnosis, and care coordination records) were collected from the medical records where available. In patients who underwent OAC treatment for at least 4 weeks, the following information was collected by the GP: ACTS, MMAS-8, and patient survey on care coordination satisfaction if they received care coordination.

### Ethical

Written, informed consent was obtained from each patient who answered patient-reported outcomes questionnaires. Data were collected from medical charts in routine medical practice and anonymized for subsequent data analysis. The opportunity to opt-out from this study was also provided through posters placed in each clinic.

The study protocol was approved collectively by the Institutional Ethics Committee/Institutional Review Board at the Research Institute for Brain and Blood Vessels-Akita on behalf of all participating clinics (approval number:15–10, approved on October 5, 2015) and the clinical research promotion network, Osaka, Japan (approved on August 27, 2015).

### Statistical analyses

In the present study, statistical analyses were of an explorative and descriptive nature. No confirmatory hypothesis testing was performed. To assess the impact of care coordination on AF treatment and the effectiveness of this campaign*,* implementation of care coordination, antithrombotic therapy patterns, and patient-reported outcomes were numerically compared between patients with and without care coordination, and between patients during the pre-campaign and campaign periods.

Baseline characteristics of AF patients were compared numerically both between those with and without care coordination and between those during the pre-campaign period and the campaign period. Any missing values or unlikely data were queried and validated. For the scoring of ACTS and MMAS-8, appropriate imputation was applied.

All statistical analyses were performed using SAS v9.4 (SAS Institute, Cary, NC, USA).

## Results

### Characteristics of newly diagnosed AF patients

There were 86 newly diagnosed AF patients during the pre-campaign period and 90 during the campaign period. The median ages of newly diagnosed AF patients during the pre-campaign period and the campaign period were 80.5 years and 82.5 years, respectively, and median CHADS_2_ scores were 2 and 3, respectively. The percentages of patients with congestive heart failure (40.0% vs. 33.7%), hypertension (75.6% vs. 74.4%), and history of stroke/transient ischemic attack (TIA) (24.4% vs. 18.6%) were higher during the campaign period than the pre-campaign period. The patients’ characteristics were comparable between the patients newly diagnosed with AF during the pre-campaign and campaign periods (Table 1).

**Table 1:** Characteristics of newly diagnosed AF patients

	Pre-campaign period *N* = 86	Campaign period *N* = 90
Male	32 (37.2)	40 (44.4)
Age, y		
Median (IQR)	80.5 (75, 87)	82.5 (76, 87)
Mean ± SD	80.8 ± 8.1	81.3 ± 7.2
Risk factors for ischemic stroke		
CHF	29 (33.7)	36 (40.0)
Hypertension	64 (74.4)	68 (75.6)
Age ≥ 75 years	67 (77.9)	72 (80.0)
Diabetes	22 (25.6)	13 (14.4)
History of stroke/TIA	16 (18.6)	22 (24.4)
CHADS_2_ score		
0	4 (4.7)	6 (6.7)
1	13 (15.1)	10 (11.1)
≥ 2	69 (80.2)	74 (82.2)
Median (IQR)	2 (2, 3)	3 (2, 3)

Data are n (%), unless otherwise stated

*Abbreviations*: *AF* Atrial fibrillation, *CHF* Congestive heart failure, *IQR* Interquartile range, *SD* Standard deviation, *TIA* Transient ischemic attack

### Implementation of care coordination

As for care coordination, 14 of 90 (15.6%) newly diagnosed AF patients were referred to cardiovascular specialists during the campaign period, and of them, 13 (92.9%) underwent care coordination. Overall, 14.4% (13/90) of the newly diagnosed AF patients received care coordination; for most of these (92.3%, *n* = 12/13), this involved referral to a cardiovascular specialist, whereas one patient was referred for specific treatment.

During the pre-campaign period, 6 of 86 (7.0%) newly diagnosed AF patients were referred to cardiovascular specialists; of them, 3 (50.0%) underwent care coordination. Overall, 3.5% (3 of the 86) of the newly diagnosed AF patients received care coordination.

When comparing the implementation of care coordination between the pre-campaign and campaign periods, the percentage of patients who underwent referral to cardiovascular specialists increased from 7.0 to 15.6% in the campaign period, and the percentage of patients who underwent care coordination increased from 3.5 to 14.4% (Table 2).

**Table 2:** Implementation of care coordination

	Pre-campaign period *N* = 86	Campaign period *N* = 90
Referral to cardiovascular specialists	6 (7.0)	14 (15.6)
Patients with care coordination	3 (3.5)	13 (14.4)

Data are n (%)

### Characteristics of patients with and without care coordination

The patient characteristics by group of patients with or without care coordination during the campaign period are shown in Table 3. In the groups of patients with or without care coordination, the median age was 79 years and 83 years, median CHADS_2_ scores were 2 and 3, the percentages of patients with history of stroke/TIA were 7.7 and 27.3%, the percentages of patients with congestive heart failure were 46.2 and 39.0%, and the percentages of patients with hypertension were 84.6 and 74.0%, respectively.

**Table 3:** Patient characteristics in patients with and without care coordination during the campaign period

Characteristic	With care coordination *N* = 13	Without care coordination *N* = 77
Male	5 (38.5)	35 (45.5)
Age, y		
Median (IQR)	79 (76, 86)	83 (76, 87)
Mean ± SD	81.1 ± 7.9	81.4 ± 7.2
Type of AF		
Paroxysmal	4 (30.8)	23 (29.9)
Persistent	5 (38.5)	30 (39.0)
Permanent	3 (23.1)	13 (16.9)
Other/unknown	1 (7.7)	11 (14.3)
Risk factor for ischemic stroke		
CHF	6 (46.2)	30 (39.0)
Hypertension	11 (84.6)	57 (74.0)
Age ≥ 75 y	11 (84.6)	61 (79.2)
Diabetes	0 (0.0)	13 (16.9)
History of stroke/TIA	1 (7.7)	21 (27.3)
CHADS_2_ score		
0	0 (0.0)	6 (7.8)
1	3 (23.1)	7 (9.1)
≥ 2	10 (76.9)	64 (83.1)
Median (IQR)	2 (2, 3)	3 (2, 3)

Data are n (%), unless otherwise stated

*Abbreviations*: *AF* Atrial fibrillation, *CHF* Congestive heart failure, *IQR* Interquartile range, *SD* Standard deviation, *TIA* Transient ischemic attack

### Antithrombotic therapy

Table 4 shows the comparison of antithrombotic therapy for stroke prevention between patients with and without care coordination during the campaign period. Overall, the status of antithrombotic therapy for stroke prevention in both groups was similar and did not differ according to the presence or absence of care coordination. The overall percentages of patients undergoing antithrombotic therapy were 84.6% vs. 76.6% in the groups with and without care coordination, respectively. The proportions of patients receiving therapy with antiplatelet agents were 15.4% vs. 10.4%, respectively, proportions receiving therapy with OACs were 69.2% vs. 68.8%, respectively, and of patients receiving therapy with OACs, the proportions of those receiving therapy with DOACs were 88.9 and 81.1%, respectively.

**Table 4:** Antithrombotic therapy in patients with and without care coordination during the campaign period

	With care coordination *N* = 13	Without care coordination *N* = 77
Antithrombotic therapy	11 (84.6)	59 (76.6)
Oral anticoagulant agents	9 (69.2)	53 (68.8)
Warfarin	1 (11.1)	10 (18.9)
DOACs	8 (88.9)	43 (81.1)
Antiplatelet agents	2 (15.4)	8 (10.4)
CHADS_2_ score low (score: 0)	*N* = 0	*N* = 6
Oral anticoagulant agents	0 (0.0)	2 (33.3)
Warfarin	0 (0.0)	0 (0.0)
DOACs	0 (0.0)	2 (100.0)
CHADS_2_ score intermediate (score: 1)	*N* = 3	*N* = 7
Oral anticoagulant agents	2 (66.7)	7 (100.0)
Warfarin	0 (0.0)	3 (42.9)
DOACs	2 (100.0)	4 (57.1)
CHADS_2_ score high (score: ≥2)	*N* = 10	*N* = 64
Oral anticoagulant agents	7 (70.0)	44 (68.8)
Warfarin	1 (14.3)	7 (15.9)
DOACs	6 (85.7)	37 (84.1)
OAC therapy based on JCS 2013 guidelines	9 (69.2)	55 (71.4)

Data are n (%)

*Abbreviations*: *JCS 2013* Japanese guidelines for pharmacotherapy of atrial fibrillation, *DOACs* Direct oral anticoagulants

The proportions of patients receiving antithrombotic therapy for stroke prevention according to the definition from the Japanese guideline for AF medication [4] did not differ between the groups, being approximately 70% in both.

The comparison of antithrombotic therapy for stroke prevention between the pre-campaign and campaign periods is shown in Table 5. More patients received antithrombotic therapy (77.8% vs. 69.8%, respectively) and were prescribed anticoagulant agents (68.9% vs. 60.5%, respectively) during the campaign period than during the pre-campaign period. Furthermore, of patients prescribed OACs, the percentage prescribed DOACs increased from 78.8% in the pre-campaign period to 82.3% during the campaign period. Of patients at low risk of stroke, the percentage of patients prescribed OACs decreased from 100% in the pre-campaign period to 33.3% during the campaign period, while among patients at intermediate and high risk of stroke, the percentage increased from 53.8 to 90.0% and from 59.4 to 68.9%, respectively. The percentage of patients who received anticoagulant therapy according to the definition from the Japanese AF medication guideline [9] increased from 55.8% in the pre-campaign period to 71.1% in the campaign period.

**Table 5:** Comparison of antithrombotic therapy between the pre-campaign and campaign periods

	Pre-campaign period *N* = 86	Campaign period *N* = 90
Antithrombotic therapy	60 (69.8)	70 (77.8)
Oral anticoagulant agents	52 (60.5)	62 (68.9)
Warfarin	11 (21.2)	11 (17.7)
DOACs	41 (78.8)	51 (82.3)
Antiplatelet agents	12 (14.0)	10 (11.1)
CHADS_2_ score low (score, 0)	*N* = 4	*N* = 6
Oral anticoagulant agents	4 (100.0)	2 (33.3)
Warfarin	0 (0.0)	0 (0.0)
DOACs	4 (100.0)	2 (100.0)
CHADS_2_ score Intermediate (score, 1)	*N* = 13	*N* = 10
Oral anticoagulant agents	7 (53.8)	9 (90.0)
Warfarin	1 (14.3)	3 (33.3)
DOACs	6 (85.7)	6 (66.7)
CHADS_2_ score high (score, ≥2)	*N* = 69	*N* = 74
Oral anticoagulant agents	41 (59.4)	51 (68.9)
Warfarin	10 (24.4)	8 (15.7)
DOACs	31 (75.6)	43 (84.3)
OAC therapy based on JCS 2013 guidelines	48 (55.8)	64 (71.1)

Data are n (%)

*Abbreviations*: *JCS 2013* Japanese guidelines for pharmacotherapy of atrial fibrillation, *DOACs* Direct oral anticoagulants

### Patient-reported outcomes

The MMAS-8 score was similar between patients with and without care coordination. The median total score for both groups was 1, and the interquartile range (IQR) was 1 to 1.5 in the group with care coordination and 0.75 to 1 in the group without care coordination. Patients in both groups had a low adherence score, regardless of care coordination (Table 6).

**Table 6:** Patient-reported outcomes in patients with and without care coordination during the campaign period

	With care coordination	Without care coordination
MMAS-8	*N* = 13	*N* = 22
Total score, median (IQR)	1 (1, 1.5)	1 (0.75, 1)
Adherence level^a^, n (%)		
Low	13 (100.0)	22 (100.0)
Medium	0 (0.0)	0 (0.0)
High	0 (0.0)	0 (0.0)
ACTS score	*N* = 14	*N* = 22
Burden		
Burden score, median (IQR)	57.5 (55, 60)	59 (57, 60)
Global question about burdens (Item 13), median (IQR)	1 (1, 2)	1 (1, 1)
Benefit		
Benefit score, median (IQR)	10.5 (9, 12)	12.5 (11, 15)
Global question about benefits (Item 17), median (IQR)	3 (3, 3)	4 (3, 5)

Use of the©MMAS is protected by US and International copyright laws. Permission for use is required. A license agreement is available from: Donald E. Morisky, MMAS Research (MORISKY) 16,636 159th Place SE, Renton WA 98058, dmorisky@gmail.com

*Abbreviations*: *MMAS-8* Morisky 8-Item Medication Adherence Scale, *ACTS* Anti-Clot Treatment Scale, *IQR* Interquartile range

^a^Adherence level: low adherence, total score < 6; medium adherence, total score 6 to < 8; high adherence, total score = 8

As for ACTS, this showed very high satisfaction, with a median burden score of 57.5 (IQR 55, 60) and 59 (IQR 57, 60), respectively, in the groups with and without care coordination. The median benefit score in the group with care coordination was slightly lower, at 10.5 (IQR 9, 12), compared with 12.5 (IQR 11, 15) in the group without care coordination (Table 6). Responses to the survey on overall satisfaction with care coordination were positive in both the pre-campaign and campaign periods (Additional file 2).

## Discussion

The present study was a multi-center, single-arm, prospective cohort study with retrospective chart review, which assessed the effectiveness of opportunistic AF screening and the impact of care coordination between GPs and cardiovascular specialists on OAC treatment for AF patients. The current report focuses on the latter objectives.

Compared with the report of the Fushimi AF Registry, a community-based survey of AF patients whose age distribution is similar to that in Japan [27], the patients in the current study had older mean age (74.2 years in the Fushimi Registry) and higher proportions of females (40.3% in Fushimi Registry) and of CHF and hypertension (27.9 and 60.6%, respectively, in The Fushimi Registry). A previous report from the Fushimi AF Registry showed that the proportions of females and of CHF and hypertension increased with age [28]. Akita prefecture is one of the super-aging regions in Japan; in 2016 [29], individuals aged ≥65 years accounted for approximately 35% of residents, with Yokote and Daisen cities showing similar percentages (36.0, and 35.4%, respectively). In addition, women accounted for 59.1, 58.8, and 59.6% of the population in Akita Prefecture and Yokote and Daisen cities, respectively.

Therefore, the characteristics of the present study’s participants reflect the specific demographics of Akita prefecture. However, since Japan is a leading super-aging society, and the percentage of the elderly population (aged ≥65 years) is expected to exceed 30% in 2025, the results of the current study could be helpful for AF management in aging regions in Japan in the future.

The results of this study indicate that this type of awareness campaign, targeted to GPs, has a role in promoting awareness of care coordination among GPs and appropriate anticoagulant therapy according to the definition from the Japanese AF medication guideline [4]. The percentage of patients receiving anticoagulant therapy according to the definition from the Japanese AF medication guideline [4] improved from 55.8% in the pre-campaign period to 71.1% in the campaign period, which was similar to the increase in OAC use (from 68 to 80%) reported in a previous study that evaluated the effectiveness of an educational campaign in patients and providers in several nations [2].

From this perspective, the campaign was successful in enhancing OAC use for stroke prevention. Implementation of care coordination in this study increased from 3.5 to 14.4%, though the percentage of care coordination implementation even after the campaign was much lower than expected. This study showed that > 80% of patients who received oral anticoagulant therapy were prescribed DOACs regardless of care coordination. DOACs, unlike warfarin, do not need INR monitoring and are easy to use in clinics, regardless of the physician’s specialty. Therefore, in this study, GPs might have been able to initiate and continue antithrombotic therapy with DOACs for their patients at their clinics and undertake stroke risk assessment without care coordination. This may have led to the lower implementation of care coordination than expected.

Several studies have evaluated the effectiveness of care coordination or integrative care in reducing hospitalizations, stroke, and death [2, 30–32]. McDonald et al. reported on the role that care coordination may play in implementing and maintaining appropriate OAC therapy in AF patients [33]. However, studies assessing the impact of care coordination in relation to OAC therapy remain limited. In the present study, an increase in OAC therapy with care coordination was not demonstrated, and OAC therapy did not differ between groups with and without care coordination. The patients’ characteristics were, in general, comparable between the groups with and without care coordination; however, it was difficult to assess the differences in individual characteristics due to there being relatively few cases of care coordination. The only difference observed between these two groups was a 4-year difference in the median age. Patients receiving care coordination were younger than those who did not receive care coordination.

There are several possible explanations for this lack of difference in prescription of antithrombotic therapies for stroke prevention between the two groups. First, considering the relationship between aging and increased permanent AF, [27] there is a possibility that younger AF patients who have not yet developed permanent AF could be referred to the cardiovascular specialist for ablation, whereas older AF patients with permanent AF likely require OAC therapy that is managed in primary care clinics. In the present study, younger patients were referred to cardiovascular specialists. Second, the lack of difference may be related to the introduction of DOACs regardless of the involvement of cardiovascular specialists. This may have a positive impact on the prescription of OACs and treatment of AF patients by GPs without care coordination, even for older patients with a higher risk of stroke. Additionally, the campaign itself may have increased awareness of and motivation to prescribe OAC therapy based on the Japanese AF medication guideline [4]. Lastly, the participating sites were volunteers; therefore, there is a possibility of bias from participating GPs toward management of AF even without the campaign.

In terms of feasibility and potential of care coordination for AF treatment, this study showed some important and unexpected characteristics of care coordination in AF treatment in Japan: implementation of care coordination seemed to be associated with each patient characteristic; approximately 70% of patients with AF without care coordination received oral anticoagulant therapy; and, of them, approximately 80% were prescribed DOACs at their GPs’ clinics. Considering these factors, there is a possibility that use of DOACs could allow patients to have the option to receive treatment at GPs’ clinics; this might decrease the elderly patients’ burden of frequent visits to hospitals for prothrombin time-international normalized ratio (PT-INR) monitoring with warfarin therapy, for example. The present findings suggest that care coordination may not be the only solution for appropriate AF treatment in the era of DOACs.

Regarding patient-reported outcomes, the ACTS was used to assess patient satisfaction with anticoagulant treatment by considering the burden and benefits of treatment [23], and adherence was assessed using the MMAS-8. Satisfaction with burden was high in both patient groups, and overall satisfaction with care coordination based on a patient survey showed a positive response. However, adherence measured with the MMAS-8 was remarkably low in both patient groups. A previous study reported that Japanese AF patients in their eighties were less adherent to DOACs prior to their development of acute ischemic stroke than those in their seventies, and it also reported beneficial effects of adequate adherence to DOACs in terms of stroke severity [34]. In this study, which had a median age of approximately 80 years, low adherence was observed for all patients. Considering these results, in aging societies as represented by Akita prefecture, improvement of adherence is a critical issue, and measures such as educational programs targeted to patients and healthcare professionals should be undertaken.

### Limitations

This study had several limitations. First, the participating primary care clinics in this study were not selected randomly, and GPs volunteered to participate. This may have introduced a selection bias and may have affected study variables such as treatment patterns, care coordination implementation, and baseline patient characteristics. Second, claims data for AF screening may not accurately match the final diagnosis in medical charts. Third, when judging whether anticoagulant therapy was based on the current guideline, data on PT-INR and creatinine clearance were not included. Fourth, owing to fewer responses to questionnaires and less implementation of care coordination than expected, it was difficult to assess the impact of care coordination. Fifth, the sample size for patient-reported outcomes was small; therefore, these results should be interpreted with caution, and further investigation is needed for more precise clarification. Lastly, this study was not designed to evaluate prognosis after care coordination. Therefore, it is impossible to evaluate the effectiveness of care coordination in terms of improvement of prognosis.

## Conclusions

To the best of our knowledge, this study is the first to evaluate the impact of care coordination between GPs and cardiovascular specialists on AF antithrombotic therapy in Japan. Overall, this study showed the positive impact of the campaign on raising the awareness of GPs regarding care coordination in the study region in Japan, with an increase in the implementation of care coordination and OAC therapy. However, there is still much room for improvement in terms of patient adherence to anticoagulants, particularly among elderly patients. While further verification of this campaign’s potential in other areas of Japan is needed, the study results show that it would be meaningful to expand this campaign model of care coordination and appropriate antithrombotic therapy into other regions.

## Supplementary information

**Additional file 1.** Primary care clinics and corresponding investigators in Daisen and Yokote, Akita, Japan. A list of the 12 primary care clinics associated with the Akita study group.

**Additional file 2.** Patients’ satisfaction with care coordination during the pre-campaign and campaign period. Table showing the results of the survey assessing patients’ satisfaction with care coordination during the pre-campaign and campaign period.

## Data Availability

The datasets used and/or analyzed during the current study are available from the corresponding author on reasonable request.

## Abbreviations

ACTS: Anti-Clot Treatment Scale
AF: Atrial fibrillation
DOAC: Direct oral anticoagulant
ESC: European Society of Cardiology
GP: General practitioners
IQR: Interquartile range
MMAS-8: Morisky 8-Item Medication Adherence Scale
OAC: Oral anticoagulant
PT-INR: Prothrombin time-international normalized ratio
TIA: Transient ischemic attack

## References

[1] Chugh SS, Havmoeller R, Narayanan K, Singh D, Rienstra M, Benjamin EJ (2014). Worldwide epidemiology of atrial fibrillation: a global burden of disease 2010 study. Circulation.

[2] Vinereanu D, Lopes RD, Bahit MC, Xavier D, Jiang J, Al–Khalidi HR (2017). A multifaceted intervention to improve treatment with oral anticoagulants in atrial fibrillation (IMPACT-AF): An international, cluster–randomised trial. Lancet.

[3] Davis RC, Hobbs FD, Kenkre JE, Roalfe AK, Iles R, Lip GY (2012). Prevalence of atrial fibrillation in the general population and in high–risk groups: the ECHOES study. Europace.

[4] JCS Joint Working Group (2014). Guidelines for pharmacotherapy of atrial fibrillation (JCS 2013). Circ J.

[5] Inoue H, Fujiki A, Origasa H, Ogawa S, Okumura K, Kubota I (2009). Prevalence of atrial fibrillation in the general population of Japan: an analysis based on periodic health examination. Int J Cardiol.

[6] Kimura K, Minematsu K, Yamaguchi T, for the Japan Multicenter Stroke Investigators’ Collaboration (J–MUSIC) (2005). Atrial fibrillation as a predictive factor for sever stroke and early death in 15,831 patients with acute ischaemic stroke. J Neurol Neurosurg Psychiatry.

[7] Wolf PA, Abbott RD, Kannel WB (1991). Atrial fibrillation as an independent risk factor for stroke: the Framingham study. Stroke.

[8] Lin HJ, Wolf PA, Kelly-Hayes M, Beiser AS, Kase CS, Benjamin EJ (1996). Stroke severity in atrial fibrillation. The Framingham study. Stroke.

[9] Benjamin EJ, Wolf PA, D'Agostino RB, Silbershartz H, Kannel WB, Levy D (1998). Impact of atrial fibrillation on the risk of death: the Framingham heart study. Circulation.

[10] Atrial Fibrillation Association (2013). 1 in 3 Brits unaware of stroke risk.

[11] Atrial Fibrillation Association. The AF report. Atrial fibrillation: preventing a stroke crisis: Atrial Fibrillation Association; 2011. http://www.preventaf-strokecrisis.org/files/files/The%20AF%20Report%2014%20April%202012.pdf. Accessed 21 Dec 2018

[12] Iwahana H, Ishikawa S, Ishikawa J, Kabutoya T, Kayaba K, Gotoh T (2011). Atrial fibrillation is a major risk factor for stroke, especially in women: the Jichi medical school cohort study. J Epidemiol.

[13] Chen MA (2016). Multimorbidity in older adults with atrial fibrillation. Clin Geriatr Med.

[14] van den Dries CJ, Oudega R, Elvan A, Rutten FH, van de Leur SJCM, Bilo HJG (2017). Integrated management of atrial fibrillation including tailoring of anticoagulation in primary care: study design of the ALL-IN cluster randomised trial. BMJ Open.

[15] Akao M, Chun YH, Esato M, Abe M, Tsuji H, Wada H (2014). Inappropriate use of oral anticoagulants for patients with atrial fibrillation. Circ J.

[16] Okumura Y, Yokoyama K, Matsumoto N, Tachibana E, Kuronuma K, Oiwa K (2017). Current use of direct oral anticoagulants for atrial fibrillation in Japan: findings from the SAKURA AF registry. J Arrhythm.

[17] Ogilvie IM, Newton N, Welner SA, Cowell W, Lip GY (2010). Underuse of oral anticoagulants in atrial fibrillation: a systematic review. Am J Med.

[18] Kirchhof P, Benussi S, Kotecha D, Ahlsson A, Atar D, Casadei B (2016). 2016 ESC guidelines for the management of atrial fibrillation developed in collaboration with EACTS. Eur Heart J.

[19] Hart RG, Pearce LA, Aguilar MI (2007). Meta-analysis: antithrombotic therapy to prevent stroke in patients who have nonvalvular atrial fibrillation. Ann Intern Med.

[20] Wagner EH, Austin BT, Von Korff M (1996). Organizing care for patients with chronic illness. Milbank Q.

[21] Nieuwlaat R, Olsson SB, Lip GY, Camm AJ, Breithardt G, Capucci A (2007). Guideline–adherent antithrombotic treatment is associated with improved outcomes compared with undertreatment in high–risk patients with atrial fibrillation. The euro heart survey on atrial fibrillation. Am Heart J.

[22] Ministry of Health, Labor and Welfare of Japan. Roundtable meeting between prefectures and counties to create a new medical plan (part 2) [in Japanese], https://www.mhlw.go.jp/shingi/2005/10/s1024-8c.html. Accessed 21 Dec 2018.

[23] Cano SJ, Lamping DL, Bamber L, Smith S (2012). The anti-clot treatment scale (ACTS) in clinical trials: cross–cultural validation in venous thromboembolism patients. Health Qual Life Outcomes.

[24] Morisky DE, Ang A, Krousel-Wood M, Ward HJ (2008). Predictive validity of a medication adherence measure in an outpatient setting. J Clin Hypertens (Greenwich).

[25] Krousel-Wood M, Islam T, Webber LS, Re RN, Morisky DE, Muntner P (2009). New medication adherence scale versus pharmacy fill rates in seniors with hypertension. Am J Manag Care.

[26] Morisky DE, DiMatteo MR (2011). Improving the measurement of self-reported medication nonadherence: response to authors. J Clin Epidemiol.

[27] Akao M, Chun YH, Wada H, Esata M, Hashimoto T, Abe M (2013). Current status of clinical background of patients with atrial fibrillation in a community-based survey: the Fushimi AF registry. J Cardiol.

[28] Yamashita Y, Hamatani Y, Esato M, Chun YH, Tsuji H, Wada H (2016). Clinical characteristics and outcomes in extreme elderly (age >/= 85 years) Japanese patients with atrial fibrillation: the Fushimi AF registry. Chest.

[29] Population and household of Akita prefecture (monthly report): October, 2016-September, 2017 [in Japanese]. https://www.pref.akita.lg.jp/pages/archive/16262. Accessed 4 July 2019.

[30] Tran HN, Tafreshi J, Hernandez EA, Pai SM, Torres VI, Pai RG (2013). A multidisciplinary atrial fibrillation clinic. Curr Cardiol Rev.

[31] Conti A, Canuti E, Mariannini Y, Viviani G, Poggioni C, Boni V (2012). Clinical management of atrial fibrillation: early interventions, observation, and structured follow–up reduce hospitalizations. Am J Emerg Med.

[32] Carter L, Gardner M, Magee K, Fearon A, Morgulis I, Doucette S (2016). An integrated management approach to atrial fibrillation. J Am Heart Assoc.

[33] McDonald KM, Sundaram V, Bravata DM, Lewis R, Lin N, Kraft SA (2007). Closing the quality gap: a critical analysis of quality improvement strategies (Vol. 7: care coordination).

[34] Yamashiro K, Kurita N, Tanaka R, Ueno Y, Miyamoto N, Hira K. Adequate adherence to direct oral anticoagulant is associated with reduced ischemic stroke severity in patients with atrial fibrillation. J Stroke Cerebrovasc Dis. 2018; [Epub ahead of print]. 10.1016/j.jstrokecerebrovasdis.2018.09.019 30318259

[35] TASK-AF. Take Action for StroKe prevention in AF [in Japanese]. http://task-af.jp/english/. Accessed 21 Dec 2018.

