# Supplementary material for: Impact of care coordination on oral anticoagulant therapy among patients with atrial fibrillation in routine clinical practice in Japan: a prospective, observational study
Source: BMC Cardiovasc Disord. 2019 Oct 24;19:235. doi: 10.1186/s12872-019-1216-y (PMC6813967; doi:10.1186/s12872-019-1216-y)
Supplement: Supplementary file 1 — Additional file 1. Primary care clinics and corresponding investigators in Daisen and Yokote, Akita, Japan. A list of the 12 primary care clinics associated with the Akita study group. [file 12872_2019_1216_MOESM1_ESM.docx]

**Additional file 1**

**Primary care clinics and corresponding investigators in Daisen and Yokote, Akita, Japan**

***Daisen City***

Kazuya Sasaki*, Sasaki Internal Medicine Clinic; Yoshiya Toyoshima*, Toyoshima Clinic; Michihiro Abe*, Ota Clinic; Noriaki Konishi*, Konishi Gastrointestinal Medicine Clinic; Sakiko Arai*, Arai Clinic; Masateru Goto*, Goto Internal Medicine Clinic; Yasukazu Kimura*, Kimura Internal Medicine Clinic and Yoshiyuki Osawa*, Osawa Gastrointestinal Medicine Clinic

***Yokote City***

Akira Takahashi*, Takahashi Internal Medicine Clinic; Tadashi Ogiwara, Ogiwara Internal Medicine Clinic; Takao Kumagai*, Kumagai Clinic; Syu Hashimoto*, Hashimoto Internal Medicine Clinic

* The Akita study group
