# Supplementary material for: Impact of care coordination on oral anticoagulant therapy among patients with atrial fibrillation in routine clinical practice in Japan: a prospective, observational study
Source: BMC Cardiovasc Disord. 2019 Oct 24;19:235. doi: 10.1186/s12872-019-1216-y (PMC6813967; doi:10.1186/s12872-019-1216-y)
Supplement: Supplementary file 2 — Additional file 2. Patients’ satisfaction with care coordination during the pre-campaign and campaign period. Table showing the results of the survey assessing patients’ satisfaction with care coordination during the pre-campaign and campaign period. [file 12872_2019_1216_MOESM2_ESM.docx]

## Additional file 2

## Patients’ satisfaction with care coordination during the pre-campaign and campaign period

| Satisfaction with care coordination | Total  (both periods)  N=16 | Pre-campaign period  N=3 | Campaign period  N=13 |
| --- | --- | --- | --- |
| Score, median (IQR) |  |  |  |
| 1. How much was the burden of seeing both a primary care physician and a cardiologist? | 1 (1, 1.5) | 1 (1, 1) | 1 (1, 2) |
| 2. How relieved did you feel about seeing both a primary care physician and a cardiologist? | 5 (4, 5) | 5 (5, 5) | 5 (4, 5) |
| 3. How satisfied are you with care coordination between the primary care physician and the cardiologist, comprehensively? | 5 (4, 5) | 5 (5, 5) | 4 (4, 5) |

Abbreviation: IQR, interquartile range

Scoring:

| Not at all | 1 |
| --- | --- |
| A little | 2 |
| Moderately | 3 |
| Quite a bit | 4 |
| Extremely | 5 |
